# The effectiveness of warning statements in reducing careless responding in crowdsourced online surveys

**DOI:** 10.3758/s13428-023-02321-z

**Published:** 2024-01-18

**Authors:** Florian Brühlmann, Zgjim Memeti, Lena F. Aeschbach, Sebastian A. C. Perrig, Klaus Opwis

**Affiliations:** Center for General Psychology and Methodology, Faculty of Psychology, University of Basel, Missionsstrasse 62a, 4055 Basel, Switzerland

**Keywords:** Data quality, Carelessness, Insufficient effort responding, Inattention, Survey methods, Warning statements

## Abstract

Carelessness or insufficient effort responding is a widespread problem in online research, with estimates ranging from 3% to almost 50% of participants in online surveys being inattentive. While detecting carelessness has been subject to multiple studies, the factors that reduce or prevent carelessness are not as well understood. Initial evidence suggests that warning statements prior to study participation may reduce carelessness, but there is a lack of conclusive high-powered studies. This preregistered randomized controlled experiment aimed to test the effectiveness of a warning statement and an improved implementation of a warning statement in reducing participant inattention. A study with 812 participants recruited on Amazon Mechanical Turk was conducted. Results suggest that presenting a warning statement is not effective in reducing carelessness. However, requiring participants to actively type the warning statement statistically significantly reduced carelessness as measured with self-reported diligence, even-odd consistency, psychometric synonyms and antonyms, and individual response variability. The active warning statements also led to statistically significantly more attrition and potentially deterred those who were likely to be careless from even participating in this study. We show that the current standard practice of implementing warning statements is ineffective and novel methods to prevent and deter carelessness are needed.

Carelessness or insufficient effort responding (IER) is a frequent problem in online research, with estimates ranging from 3 to 9% (Maniaci & Rogge, 2014), over 10–12% (e.g., Meade & Craig, 2012), and 18–27% (Peer et al., 2017) up to 45.9% (Brühlmann et al., 2020) of participants in surveys exhibiting such behavior. IER can have serious consequences such as failed manipulations (Oppenheimer et al., 2009), false-positive findings (Huang et al., 2015), and unfavorable psychometric properties of scales (Johnson, 2005; Maniaci & Rogge, 2014). Thus, ensuring quality data is vital for on- and offline research. The prevalence of IER in online surveys seems to depend on sample characteristics and the detection methods employed (Curran, 2016; Toich et al., 2021). While much work has focused on identifying and excluding participants exhibiting IER, there are still many open questions on how to effectively reduce IER in online surveys (see e.g., Arthur et al., 2021). There are many influencing factors over which researchers have little control, such as environmental distractions (Carrier et al., 2009), participant–researcher distance (Meade & Craig, 2012), or participant multitasking (Zwarun & Hall, 2014). However, one frequently used technique aiming to reduce carelessness is to make participants aware of the consequences of inattentive responding. For instance, researchers may tell participants that they will withhold incentives if participants respond inattentively or, alternatively, emphasize the importance of valid responses and the meaning of the study.

In Berinsky et al. (2016), a warning statement improved passage rates compared to respondents who went through the survey without a warning by eight percentage points. Huang et al. (2012) found that warning statements reduce the severity of IER. Similarly, Breitsohl and Steidelmüller (2018) demonstrated that informing participants that methods to assess carefulness are used improved scale reliability in a survey. They used the same wording as Huang et al. (2015), who also found positive effects on self-reports from participants when using a warning statement. Bowling et al. (2020) found that participants in a warning condition, compared to a control group, began engaging in careless responding considerably later in the survey. In contrast to the aforementioned studies, Meade and Craig (2012) found that warning statements provided no benefit over using identified responses without the warning and decreased respondent self-reported attitude toward the study. More recently, Toich et al. (2021) found no statistically significant differences between negative and positive warning statements as a means to reduce IER in Amazon’s Mechanical Turk (MTurk) and University Participant Pool samples.

Taken together, there seems to be slightly more evidence for the effectiveness of warning statements to deter insufficient effort responding. In their review, Ward & Meade (2023) concluded that using more positive approaches such as offering rewards for careful responding, have been less effective than warnings. However, the implementation of these warning statements differed substantially across studies, and it is unclear how warnings affect various carelessness detection methods. In Berinsky et al. (2016), participants were informed that each of their responses will be checked and only responses that show that participants have read and understood the survey would be accepted. Huang et al. (2012) mentioned that sophisticated statistical control methods would be used to check the validity of responses and that participants could lose their credits if they are not being attentive. This wording was also used in a slightly adapted form in Bowling et al. (2020). Similarly, but without naming any consequences, Huang et al. (2015) and Breitsohl and Steidelmüller (2018) instructed participants that several methods to assess carefulness and to ensure data quality were used. In the stern warning condition, Meade and Craig (2012) emphasized the importance of the responses and that honesty and thoughtful responses are subject to academic integrity policy.

Thus, it seems that warning statements have the potential to reduce IER, but the concrete implementation of these statements, especially concerning the consequences of inattentive responding, varies greatly between studies. To date, there is no comprehensive study that examines the effects of warning statements on various carelessness detection methods with preregistered hypotheses. Therefore, this study aims to test the general hypothesis that warning statements reduce carelessness in a registered randomized controlled experiment with two variants of warning statement implementations.

## Purpose of the present study

In the present study, we examined the effectiveness of warning statements on nine IER measures for participants in an online survey on MTurk. The preregistered experiment was designed in line with the works by Huang et al. (2012), Huang et al. (2015), and Toich et al. (2021), using the 300 items of the International Personality Item Pool-NEO inventory (IPIP-NEO; Goldberg et al., 2006; Goldberg, 1999). The effectiveness of a warning statement with the potential for negative consequences (loss of reimbursement for participation) was tested in a passive and an active condition. In the passive condition, the warning statement was *presented* to participants before the main part of the questionnaire, while in the active condition, participants were required to write down the statement in a text box, to ensure attentive reading of the statement. Most studies that examined the effects of warning statements merely presented the statement and did not require participants to demonstrate that they had read the warning. We expected that writing down, as a way to increase engagement with the warning statement, makes the warning more likely to be remembered while completing the survey and that this condition would maximize the effectiveness of the warning statement. Thus, we expected that participants who were asked to read the warning statement and those who were additionally required to write down the statement to show less carelessness throughout the survey than in the control condition.

To detect IER, we selected nine methods of those recommended in Curran (2016) and Huang and Wang (2021) namely attention check items (instructed response items, bogus/infrequency items), self-report items, and response time as procedural intervention indices and longstring, Mahalanobis distance, even-odd consistency, psychometric synonym and antonym, and individual response variability as post hoc statistical indices. In line with recommendations in Meade & Craig (2012) and Ward & Meade (2023), we used several different carelessness indices to be able to capture multiple forms of inattentive responding. However, analyzing the effects of the three conditions on nine different carelessness methods may never show a unified picture where conditions differ on all or on none of the methods. Therefore, we aim to answer the following research question:

Research question: Do warning statements reduce participant inattention, as measured by the rate of failed attention check items (instructed response items, bogus items/infrequency items) and the values on self-report items, response time, longstring, Mahalanobis distance, even-odd consistency, psychometric synonym and antonym, and individual response variability?

We propose the following hypotheses (H1a–H9b):

For the two warning statement conditions, we expect, in general, participants in the active condition to show less inattentive behavior than those in the passive condition. We do not expect this effect to be large enough to reach statistical significance.

## Preregistration

The confirmatory hypotheses H1a to H9b were registered on OSF (https://osf.io/jeurs/) prior to data collection.

## Reporting

We report how we determined our sample size, all data exclusions, all manipulations, and all measures in the study.

## Method

### Power analysis

We conducted an a priori power analysis with the effect sizes reported in Table 1 in Huang et al. (2012). The effect sizes ranged from *d* = 0.13 for response time up to *d* = 0.43 for individual reliability. Using the smallest observed effect size of *d* = 0.13 (Cohen’s *f* = 0.065), α = 0.05, and a power of 1-beta = .95, a sample size of 1219 per group (one-way ANOVA) returned. However, a sample size of 3657 was beyond the budget of this research. The funds were limited to a sample of 810 participants in total (about CHF 2000 including fees). Thus, a sensitivity analysis revealed that with an *N* of 270 per group and a power of 1-beta = .95, a small to medium effect could be detected, *f* = 0.1383, *d* = 0.277. With a lower power of 1-beta = .80, and *N* = 270, a small effect of *f* = 0.1093, *d* = 0.216, can be studied. All power analyses were conducted using G*Power 3.1.9.6 (Faul et al., 2009).

**Table 1:** Preregistered hypotheses

No.	Hypothesis
Procedural intervention indices	
H1a	The number of failed attention check items (instructed response items, bogus items/infrequency items) is significantly lower in the active warning condition than in the control condition.
H1b	The number of failed attention check items (instructed response items, bogus items/infrequency items) is significantly lower in the passive warning condition than in the control condition.
H2a	The average self-reported diligence is significantly higher in the active warning condition than in the control condition.
H2b	The average self-reported diligence is significantly higher in the passive warning condition than in the control condition.
H3a	The average page response time is significantly longer in the active warning condition than in the control condition.
H3b	The average page response time is significantly longer in the passive warning condition than in the control condition.
Post hoc statistical indices	
H4a	The average longstring is significantly lower in the active warning condition than in the control condition.
H4b	The average longstring is significantly lower in the passive warning condition than in the control condition.
H5a	The average Mahalanobis distance is significantly lower in the active warning condition than in the control condition.
H5b	The average Mahalanobis distance is significantly lower in the passive warning condition than in the control condition.
H6a	The average even-odd consistency is significantly higher in the active warning condition than in the control condition.
H6b	The average even-odd consistency is significantly higher in the passive warning condition than in the control condition.
H7a	The average correlations of psychometric synonyms is significantly higher in the active warning condition than in the control condition.
H7b	The average correlations of psychometric synonyms is significantly higher in the passive warning condition than in the control condition.
H8a	The average correlations of psychometric antonyms is significantly lower in the active warning condition than in the control condition.
H8b	The average correlations of psychometric antonyms is significantly lower in the passive warning condition than in the control condition.
H9a	The average individual response variability is significantly higher in the active warning condition than in the control condition.
H9b	The average individual response variability is significantly higher in the passive warning condition than in the control condition.

### Sample

As Agley et al. (2022), Huang et al. (2015), and Toich et al. (2021), we used the crowdsourcing service MTurk to recruit a self-selected sample of participants for this study. Participants were compensated with $1.82 for an estimated 15 min of survey time based on the US federal minimum wage of $7.25/h. To be eligible, participants had to be at least 18 years of age and a resident of the United States. No other inclusion or exclusion criteria were applied on MTurk; participants with low numbers of completed and approved HITs were allowed to participate. Based on the power analysis, a sample of 810 people was targeted. Thus, the stopping rule was defined as follows: Data collection will stop when a sample of 810 non-duplicate responses is reached, which exhausts the research budget.

### Instrument

Three procedural intervention indices and six post hoc statistical indices were used to detect careless responding. All indices are presented below.

#### Attention checks

To identify inattentive respondents, attention check items, such as instructed response items (IRI) and infrequency items is frequently recommended (IF; Curran, 2016, Maniaci & Rogge, 2014, Ward & Meade, 2023)*.* Three instructed response items were included in the survey. These items instruct participants to select a predefined response. The instructions in this survey were “Please leave this item blank”, “Please select 'Moderately Inaccurate' for this item”, and “Please select 'Moderately Accurate' for this item (Brühlmann et al., 2020; DeSimone et al., 2015. For each participant, the number of incorrect responses was used for analysis. Three infrequency items, also referred to as bogus items, from Huang et al. (2015) were included in the survey. Such infrequency items ask participants about most unlikely actions or behavior. The infrequency items in this study were ‘‘I eat cement occasionally,’’ ‘‘I can teleport across time and space,’’ and ‘‘I have never used a computer’’ (Huang et al., 2015). Both the infrequency items and the instructed response items were randomly allocated to one of the six pages, so that each page included one of each type of item. Participants were asked to indicate their level of agreement from very inaccurate (1) to very accurate (5). Responses with (4) ‘Moderately Accurate’ or (5) ‘Very Accurate’ were flagged as inattentive. The number of flags per participant was used for analysis.

#### Self-report diligence

Nine self-report diligence items (Meade & Craig, 2012) were included at the end of the survey. The series consisted of five true-keyed (e.g., “I carefully read every survey item”) and four false-keyed items (e.g., “I was dishonest on some items”). As with the infrequency items, participants were asked to rate the self-report diligence items from very inaccurate (1) to very accurate (5). After inverting the scores on the false-keyed items, the average of these nine items was used for analysis. Reliability was high with Cronbach’s α = .84, 95% CI [.82, .86], and McDonald's ω = .86, 95% CI [.85, .88].

#### Response time

Response time per page is by default recorded by the survey software used in this study. Response time can be a valuable measure of participant carelessness, but no agreed-upon cutoff exists (Curran, 2016). Therefore, we averaged the response time for each page of the IPIP inventory for further analysis. To approximate the distribution of the variable to a normal distribution, we used the log of response time for further statistical analyses.

#### Mahalanobis distance

The Mahalanobis distance is a statistical multivariate outlier analysis that “compares a respondent’s scores to the sample mean scores across all items within a survey” (DeSimone et al., 2015, Mahalanobis, 1936). Moreover, it estimates the multivariate distance between the sample mean of each measure and a participant's responses on the survey items (DeSimone et al., 2015, Mahalanobis, 1936). Hence, Mahalanobis distance allows to detect participant’s careless response behavior when examining the multivariate outliers in each participant's responses due to the assumption that participants responding with insufficient effort present an extreme deviation from normal response patterns (DeSimone et al., 2015). For each participant, the Mahalanobis distance with the 300 items of the IPIP inventory was calculated and used for analysis.

#### Longstring

An indication for low-quality data are participants' response patterns (i.e., repeated selection of same options) that can be checked using the longstring index (DeSimone et al., 2015). Moreover, consecutive identical responses might display careless responses. The longstring finds application when long surveys with a variety of multidimensional constructs or a mix of positively and negatively worded items are implemented in the survey (DeSimone et al., 2015; Huang and Wang, 2021). The average longstring for each participant was used for further analysis.

#### Even-odd consistency

This index has been termed by Meade and Craig (2012) and is also known as individual reliability (Jackson, 1976). This method measures unidimensional scales divided by using an even-odd split (Meade & Craig, 2012). Furthermore, a within-person correlation is then conducted based on the split subsets for each, the even and the odd scale.

#### Psychometric synonym/antonym

These post hoc methods were implemented following Meade and Criag (2012), meaning that a threshold of .60 was used for identifying item pairs with positive correlations for the PS index. This resulted in 352 item pairs. However, no item pairs with correlations below – .60 could be identified for the psychometric antonym index. Instead, we had to deviate from the preregistration and decided to set the threshold at -.144, resulting in 30 item pairs for the analysis, which is the same number of items used by Johnson (2005).

#### Individual response variability

Dunn et al. (2018) introduced the individual response variability index as a more robust extension of the longstring index. The index is calculated as the within-individual standard deviation of responses represented by item-sets across different constructs before rescoring reversed-coded items (Dunn et al., 2018).

### Design

This study was implemented on the online survey platform EFS Unipark^1^. Participants provided informed consent on the first page of the survey; next, they were asked about their age and gender. After the initial demographic survey, participants were randomly allocated to one of the three experimental conditions (see Table 2). In the active warning condition, we asked participants to copy the exact statement into a text area below. We included the statement as an image to prevent simple copy and paste. Thus, participants could not select the text and had to type in the statement. A custom script checked whether the provided text matched with the intended statement. We allowed a small margin of error such as incorrect punctuation or minor spelling errors. Once participants wrote down 98% of the statement, the “continue” button became available. The intention was that participants had to actively engage with the warning statement to improve the strength of the experimental manipulation (i.e., reducing inattention by increasing the salience of consequences). In the passive warning condition, we informed participants that there were mechanisms in place to detect carelessness. We chose to employ for both conditions the same statement as in Toich et al. (2021) for two reasons: First, the statement warns of comparatively severe consequences, which are more directly and stronger worded than in other works (e.g., Berinsky et al., 2016; Brink et al., 2019). Thus, we were aiming to maximize the potential deterrence effect of warning statements. Second, the statement is more appropriate in the context of crowdsourcing than other wordings, such as those in Meade and Craig (2012) ; Huang et al. (2015). In the control condition, we simply stated that the survey begins on the next page.

**Table 2:** Statement used in the experimental conditions

Condition	Instruction
Active Warning	Please read the following information carefully and copy the text into the text area below. IMPORTANT: This survey contains various mechanisms used to catch those who respond carelessly or randomly to the survey questions. If you are caught doing so, you will NOT be given credit for your participation and may be subject to additional penalties.
Passive Warning	Please read the following information carefully. The survey questions will begin on the following page. IMPORTANT: This survey contains various mechanisms used to catch those who respond carelessly or randomly to the survey questions. If you are caught doing so, you will NOT be given credit for your participation and may be subject to additional penalties.
Control	The survey questions will begin on the following page.

**Fig. 1:**
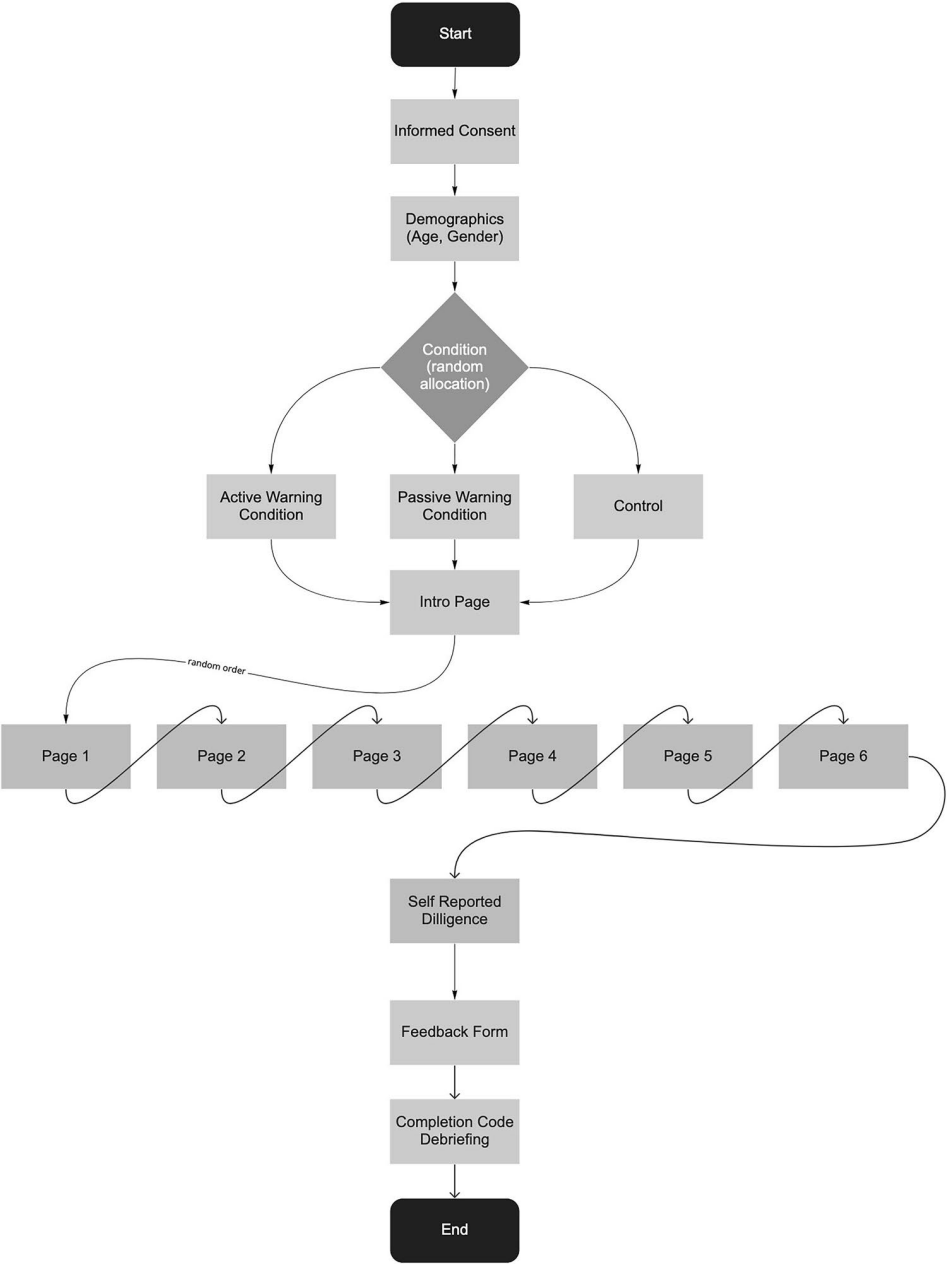
Participant flow chart

After displaying the different statements, we presented a total of 306 items over six pages to the participants. Three hundred items stem from the original International Personality Item Pool-NEO inventory (IPIP-NEO; Goldberg et al., 2006; Goldberg, 1999). To detect IER in this part of the survey, we used three instructed response items (Brühlmann et al., 2020; DeSimone et al., 2015) and three infrequency items (Huang et al., 2015). Thus, for each of the six pages, 50 items of the IPIP-NEO and 1 instructed or infrequency item were chosen randomly prior to the study. In the end, each page consisted of a total of 51 items with the addition of one instructed response item per page. These items were rated on a five-point Likert scale from very inaccurate (1) to very accurate (5). All the items within each page and the order of the six different pages were randomized. Due to technical limitations of the survey platform, items were not randomized across different pages. The order of the items and pages presented was saved for each person, this order was used to sort the items for the longstring analysis. Upon completion of this section of the study, we presented participants with nine self-reported diligence items (Meade & Craig, 2012). Lastly, we provided the opportunity to give feedback and then presented a completion code for MTurk. The survey was designed to prevent participants from skipping questions (apart from the instructed response items). The participant flow of the study is depicted in Fig. 1.

### Analytic strategy

By convention, we set the significance level to α = .05. Hypotheses 1a and 1b were tested using a negative binomial regression model. For all other hypothesis tests, we run one-way ANOVAs with planned contrasts for the hypotheses variants a and b. For none of the dependent variables, all assumptions of the ANOVA (i.e., no severe outliers, normal distribution of the residuals, homogeneity of variances) were met. Therefore, we decided to confirm all results using Kruskal–Wallis tests with Dunn’s test of multiple comparisons for the planned contrasts. When the results of the nonparametric tests differed from parametric ones, both are reported in the manuscript. Analyses were performed with R version 4.2.2 (R Core Team, 2022).

## Results

Of a total of 913 complete responses, 97 were identified as duplicate responses and four were not from the United States. These responses were removed, and a final sample of 812 participants remained. The participants were unevenly distributed to the three conditions (χ^2^ = 9.668, *df* = 2, *p* = .008): There were 289 in the control condition, 294 in the passive warning condition, and 229 in the active warning condition. Participants abandoned the study more frequently when they saw that they were required to write down a text in the active warning condition. In the active warning condition, participants spent more time writing down a text (M = 5.06 min, Mdn = 3.35 min, SD = 6.04 min) than participants who read the warning statement (M = 15.6 s, Mdn = 8 s, SD = 27.1 s) and participants in the control condition (M = 7.99 s, Mdn = 4 s, SD = 27.5 s). The majority were women (*n* = 421, 51.8% and *n* = 3, 0.3% non-binary or self-described) and on average 34.8 years old (SD = 10.4). Participants spent more time completing the survey in the active warning condition (M = 19.8 min, Mdn = 16.9 min, SD = 10.5 min) than in the passive warning condition (M = 15.8 min, Mdn = 13.2 min, SD = 9.76 min) and the control condition (M = 15.3 min, Mdn = 12.8 min, SD = 8.93 min). The overall median completion time was 14 min and 14.52 s. In the following, we present results in the order of the hypotheses in Table 1.

### Attention check items (H1a and H1b)

Between 37.1% and 48% of the participants missed one or more of the six attention check items. Descriptively, in the active warning condition, participants were more likely to spot the attention check items and respond correctly (62.9%, see Table 3). Thus, a negative binomial model was used to examine the relation of condition and the number of flags on the attention check items. Overall, the variable condition was not a significant predictor of the number of flags, LR = 1.765, *df* = 2, *p* = .414. Using dummy-coding for the variable condition, the predictor condition-active-warning was not significant, B = – 0.1038, SEB = 0.1706, *p* = .543, expB = 0.901, 95% CI [0.645, 1.248]. Likewise, condition-passive-warning was also not a significant predictor in the model, B = 0.1164, SEB = 0.1542, *p* = .450, expB = 1.123, 95% CI [0.831, 1.521]. Thus, hypotheses H1a, that the number of failed attention check items is lower in the active warning condition than in the control condition, and H1b, that the number of failed attention check items is lower in the passive warning in comparison to the control condition, could not be supported.

**Table 3:** Number of flagged attention check items (sum of instructed response and infrequency items) for each condition

Number of flags	Control (*N* = 289)		Passive warning (*N* = 294)		Active warning (*N* = 229)	
	*N*	%	*N*	%	*N*	%
0	159	55.0%	153	52.0%	144	62.9%
1	56	19.4%	61	20.7%	40	17.5%
2	48	16.6%	42	14.3%	23	10.0%
3	18	6.2%	21	7.1%	9	3.9%
4	4	1.4%	7	2.4%	9	3.9%
5	4	1.4%	9	3.1%	2	0.9%
6	0	0.0%	1	0.3%	2	0.9%

#### Additional procedural intervention indices (H2a to H3b)

**Fig. 2:**
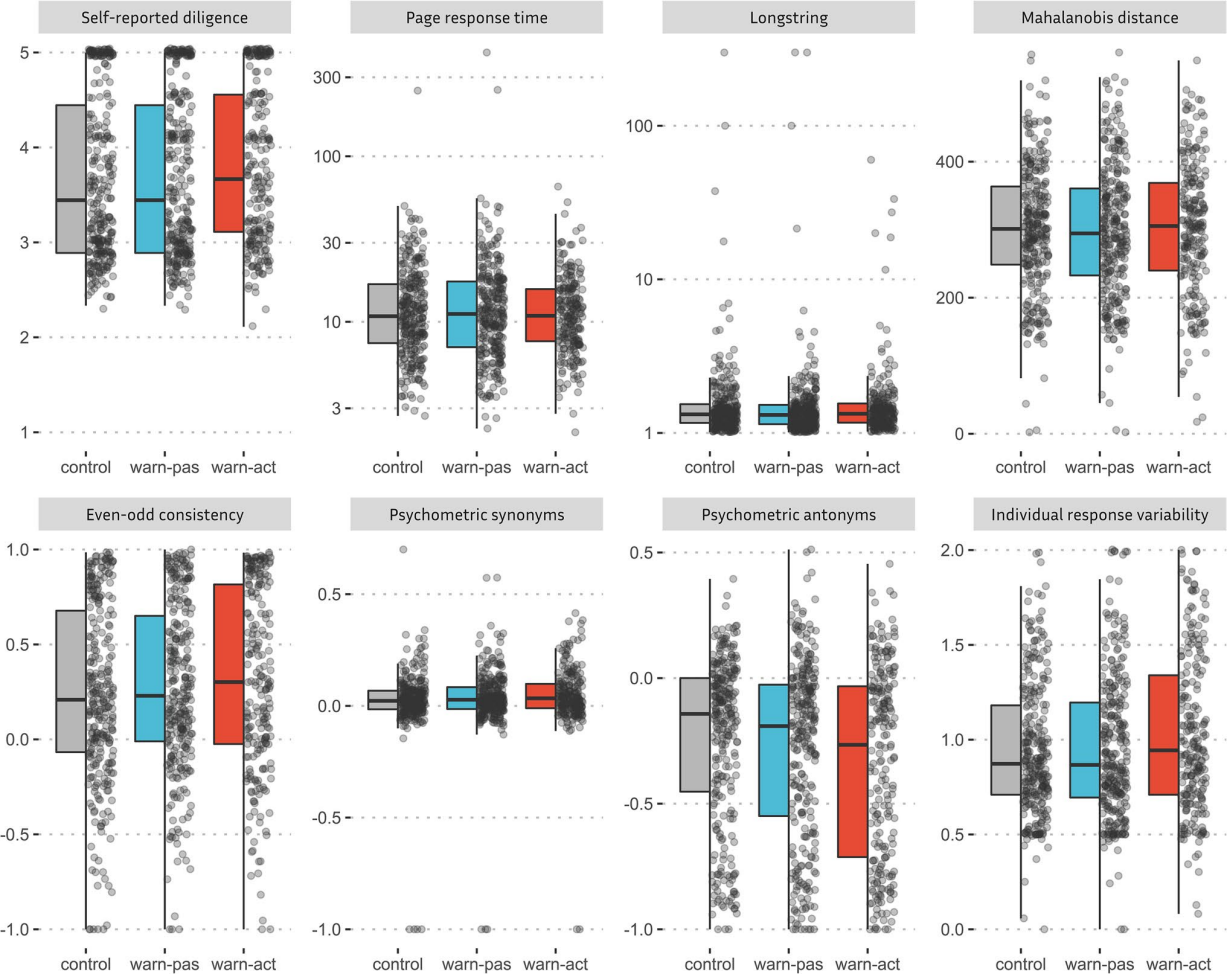
Boxplots and distribution of all interval-scaled carelessness indices. *Note*. warn-pas = passive warning condition; warn-act = active warning condition

Refer to the upper part of Table 4 for descriptive statistics on the procedural intervention indices *self-reported diligence* and *page response time*. Boxplots and distributions of these indices are depicted in Fig. 2.

**Table 4:** Descriptive statistics for all interval-scaled carelessness indices

Index	Condition	M	Mdn	SD	*d* [95% CI d]
Procedural intervention indices					
Self-reported diligence	Control	3.674	3.444	0.857	
	Active warning	3.827	3.667	0.846	0.180, [0.006, 0.354]
	Passive warning	3.69	3.444	0.854	0.020, [– 0.143, 0.182]
Page response time	Control	14.066	10.8	16.221	
	Active warning	12.667	10.867	7.946	– 0.069, [– 0.242, 0.105]
	Passive warning	15.627	11.15	29.043	0.076, [– 0.086, 0.239]
Post hoc statistical indices					
Longstring	Control	3.062	1.322	18.615	
	Active warning	2.171	1.333	5.044	0.047, [– 0.127, 0.221]
	Passive warning	3.871	1.31	25.246	– 0.043, [– 0.205, 0.120]
Mahalanobis distance	Control	300.414	301.141	91.985	
	Active warning	301.015	305.375	97.982	– 0.006, [– 0.180, 0.167]
	Passive warning	297.783	294.547	98.467	0.027, [– 0.135, 0.190]
Even-odd consistency	Control	0.237	0.209	0.474	
	Active warning	0.316	0.302	0.491	0.168, [– 0.005, 0.342]
	Passive warning	0.274	0.23	0.446	0.0798, [0.083, 0.242]
Psychometric synonyms	Control	0.024	0.023	0.149	
	Active warning	0.047	0.034	0.139	0.159, [0.015, 0.333]
	Passive warning	0.035	0.028	0.141	0.077, [0.086, 0.239]
Psychometric antonyms	Control	– 0.244	– 0.142	0.332	
	Active warning	– 0.334	– 0.266	0.38	0.253, [0.078, 0.428]
	Passive warning	– 0.281	– 0.191	0.359	0.105, [– 0.059, 0.268]
Individual response variability	Control	0.945	0.873	0.352	
	Active warning	1.025	0.944	0.413	0.213, [0.039, 0.387]
	Passive warning	0.955	0.867	0.375	0.027, [– 0.136, 0.189]

**Fig. 3:**
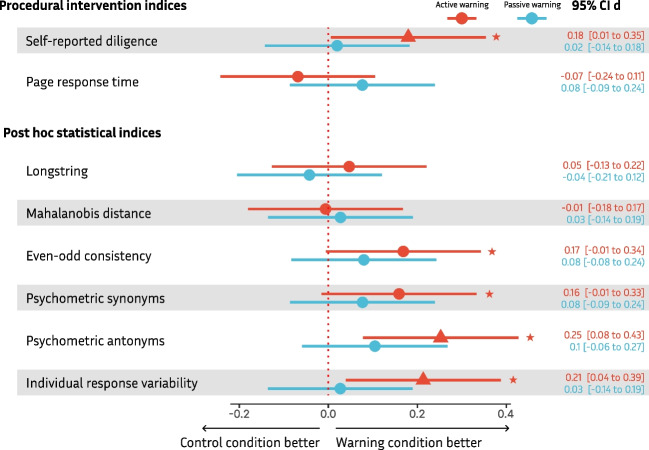
Overview of the differences in Cohen’s *d* with 95% confidence intervals between the control and warning statement conditions across all indices except attention checks. *Note*. *Asterisks* indicate statistically significant differences between control and warning statement conditions with *p* < .05 using parametric contrasts with one-sided tests. *Triangles* indicate that these differences were also statistically significant with nonparametric Dunn tests. For page response time, better means longer response time

Results are in favor of H2a, the active warning statement increased self-reported diligence, but not in favor of H2b, the passive warning did not increase diligence in comparison to the control condition (see Table 5 and Fig. 3). However, no significant differences between the conditions were observed for page response time (H3a and H3b).

**Table 5:** Results of statistical tests for hypotheses H2a to H9b

Index	H	Omnibus test	Direction	Parametric contrasts	Nonparametric Dunn test
Procedural intervention indices					
Self-reported diligence	H2a	F(2, 809) = 2.39, *p* = .092	Active warning > control	*t*(809) = 2.030, ***p* = .0213**	*z* = 2.183, ***p* = .029**
	H2b		Passive warning > control	*t*(809) = 0.239, *p* = .406	*z* = 0.146, *p* = .884
Page response time (log)	H3a	F(2, 809) = 0.30, *p* = .737	Active warning < control	*t*(809) = – 0.536, *p* = .403	*z* = – 0.365, *p* = .715
	H3b		Passive warning > control	*t*(809) = 0.247, *p* = .704	*z* = 0.119, *p* = .905
Post hoc statistical indices					
Longstring	H4a	F(2, 809) = 0.52, *p* = .597	Active warning > control	*t*(809) = 0.530, *p* = .298	*z* = 0.384, *p* = .701
	H4b		Passive warning < control	*t*(809) = – 0.513, *p* = .696	*z* = – 1.204, *p* = .228
Mahalanobis distance	H5a	F(2, 809) = 0.09, *p* = .916	Active warning < control	*t*(809) = – 0.071, *p* = .528	*z* = – 0.167, *p* = .867
	H5b		Passive warning > control	*t*(809) = 0.331, *p* = .371	*z* = 0.449, *p* = .653
Even-odd consistency	H6a	F(2, 809) = 1.82, *p* = .163	Active warning > control	*t*(809) = 1.904, ***p* = .029**	*z* = 1.908, *p* = .056
	H6b		Passive warning > control	*t*(809) = 0.963, *p* = .168	*z* = 0.890, *p* = .373
Psychometric synonyms	H7a	F(2, 809) = 1.62, *p* = .199	Active warning > control	*t*(809) = 1.796, ***p* = .036**	*z* = 1.750, *p* = .080
	H7b		Passive warning > control	*t*(809) = 0.928, *p* = .177	*z* = 0.759, *p* = .448
Psychometric antonyms	H8a	F(2, 802) = 4.05, *p* = .018	Active warning > control	*t*(802) = 2.844, ***p* = .002**	*z* = 2.53, ***p* = .011**
	H8b		Passive warning > control	*t*(802) = 1.258, *p* = .104	*z* = 1.391, *p* = .164
Individual response variability	H9a	F(2, 809) = 3.33, *p* = .036	Active warning > control	*t*(809) = 2.411, ***p* = .008**	*z* = 2.011, ***p* = .044**
	H9b		Passive warning > control	*t*(809) = 0.324, *p* = .373	*z* = 0.142, *p* = .887

Page response time was log-transformed for analysis

#### Post hoc statistical indices (H4a to H9b)

The lower part of Table 4 presents descriptive statistics for each of the six post hoc statistical indices *longstring*, *Mahalanobis distance, even-odd consistency, psychometric synonyms, psychometric antonyms,* and *individual response variability*. Distributions and boxplots of these indices are presented in Fig. 2.

Statistical analyses showed significant differences between the active warning and the control condition for psychometric antonyms and individual response variability. Thus, hypotheses H8a and H9a were supported. Additionally, using parametric a priori contrasts, participants in the active warning condition also showed significantly higher even-odd consistency and correlation of psychometric synonyms than the control condition. However, these differences were not statistically significant using Dunn’s nonparametric test, therefore, only partial support for H6a and H7a was found. For the other hypotheses we could not find statistical significance using either parametric a priori contrasts or Dunn's nonparametric test. Refer to Table 5 for an overview of the statistical analyses and their results.

### Summary of results

In summary, the passive warning condition (hypotheses variants b) did not show an improvement over the control condition in any of the indices. In the active warning condition, significantly more participants dropped out than in the other conditions once they realized that they were required to write a text. However, of those remaining, a statistically significant reduction in carelessness was observed in self-reported diligence, even-odd consistency, psychometric synonyms, psychometric antonyms, and individual response variability in comparison to the control condition (hypotheses variants a). Refer to Fig. 3 for an overview for the effect size differences between warning conditions and the control condition.

## Discussion

Survey data are increasingly collected online and ensuring the quality of the collected data has become an ever-growing concern for researchers (Ward & Meade, 2023). Preventing carelessness before it occurs is preferable to detecting careless respondents and extensive data cleaning procedures. Carelessness in responding to surveys becomes especially important in the case of utilizing a crowdsourced sample, as participants stemming from this sample are compensated monetarily for their responses. Further, this sample is aware of commonly used carelessness detection methods due to their experience in taking surveys. Warning respondents that their responses will be screened for quality and that they may not be compensated for low-quality responses is a commonly used and straightforward prevention method. Various works have shown that such warning statements prior to study participation can reduce carelessness (Berinsky et al., 2016; Breitsohl & Steidelmüller, 2018; Huang et al., 2012; Huang et al., 2015). In a recent review, Ward & Meade (2023) suggested that warning statements could be more effective than informational statements (e.g., about the important contribution to research) in reducing inattentive behavior. However, crowdsourced workers may also be deterred by these warning statements, due to their previous experience. Additionally, other studies have provided no evidence that such warnings could work (e.g., Meade & Craig, 2012). Thus, there is a need to investigate whether such warnings effectively reduce all or some forms of careless responding with preregistered hypotheses and high statistical power.

In the present study, we aimed to fill this gap by studying the effect of warning statements in reducing careless responding in a sample recruited on MTurk. However, we could not replicate the prevention effect of presenting warning statements on the three procedural intervention indices (i.e., self-reported diligence, page response time, and attention check items). Even more, none of the six post hoc statistical indices (i.e., longstring, Mahalanobis distance, even-odd consistency, psychometric synonyms/antonyms, and individual response variability) showed statistically significant improvements for the passive warning condition (i.e., presenting a warning text) compared to the control condition. This finding is important because researchers often implement warnings about the potential loss of compensation into their surveys in the hope that it will dissuade careless behavior (e.g., in Agley et al., 2022). However, results from our study show that they are ineffective in reducing participant carelessness. A plausible explanation is that participants frequently skip such instructions and, thus, do not process the information presented there. It may also be that the non-naïve workers on Mechanical Turk (cf., Chandler et al., 2014) have become accustomed to such warnings, which reduces their potential effectiveness. Thus, presenting a comparatively strongly worded warning had no preventive effect in a sample recruited on MTurk. To account for careless participants often not reading such instructions or statements (e.g., see Oppenheimer et al., 2009), we required them to copy down the warning into a text area in the active warning statement condition. With this procedure, we expected participants to process the statement more thoroughly and, consequently, we should have been able to observe a more substantial effect of this warning on the carelessness indices.

In general, our results support these considerations. Copying down the warning statement into a text area had a positive impact on participant carelessness, specifically in reducing psychometrically inconsistent responding, increasing response variability, even-odd consistency, and self-reported diligence. In contrast, merely presenting a warning statement in the passive condition resulted in no statistically significant improvement. However, it is also important to note that the active warning condition resulted in statistically significantly higher participant attrition compared to the passive warning condition, suggesting that the positive impact on various carelessness indices might be because potentially careless participants were deterred by this method. In the following, we will discuss results for each carelessness index investigated in this study.

Attention check items have the advantage of relatively little ambiguity in scoring, meaning that, for instance, the absence of the instructed response or selecting an impossible or very unlikely response on an infrequency item, are strong indicators of careless responding (Meade & Craig 2012, Ward & Meade, 2023). In our study, participants were not less likely to miss attention check items in the two warning conditions than in the control condition. Descriptively, however, in the active warning condition, more participants were not flagged by any of these check items. Although workers on MTurk are experienced with attention check items (Hauser & Schwarz, 2016), still between 37.1% and 48% of the participants in each condition were flagged by at least one of the attention check items. This suggests that attention check items are still a helpful method for unambiguously detecting careless responding on MTurk. However, the warning statements were ineffective in increasing participants' attention to these items. The same participants who miss responding instructions in items may also skip or disregard warning statements.

We further assessed self-reported diligence, which was statistically significantly higher in the active warning condition than in the control condition (*d* = 0.18). This does not necessarily mean that these participants were answering more diligently, but it does point to the active warning condition potentially facilitating a heightened experience of diligence. A potential consequence of increased diligence in responding to the items of questionnaires is longer response time, since more in-depth processing of the questionnaire would require more time. However, we observed no statistically significant differences in page response time between the conditions.

The indices designed to identify respondents that are multivariate outliers (Mahalanobis distance) or provide a high number of identical answers (longstring), did not differ significantly between the conditions. However, participants responded more consistently to psychometric synonyms (*d* = 0.16) and antonyms (*d* = 0.25) and to the even and odd items (*d* = 0.17) of the IPIP-NEO in the active warning condition than in the control condition. This shows that the active warning was successful in increasing the consistency of the responses, both in relation to the whole sample (psychometric indices), as well as to the content of the scale (even-odd index). Furthermore, participants in the active warning condition also showed greater response variability as measured by the individual response variability index (*d* = 0.21). However, this result should be interpreted with caution, because individual response variability may be affected by overly consistent or random responding similarly. Thus, conceptually, greater response variability could also mean more carelessness.

From our study, we do not yet know whether the observed positive effects in the active warning condition are due to the content of the warning statement or the fact that participants were required to write down a statement. This is because writing down the statement in the active warning condition required more time and effort than only reading a warning statement. Similarly to instructional manipulation checks in study 2 by Oppenheimer et al. (2009), the active warning condition required active engagement and correct response to proceed with the survey. The unequal distribution of participants to the three conditions shows that more participants abandoned the survey when they realized that they had to write down a statement. Thus, it may be that the active warning statement led potentially careless participants to abandon the study, that the warning increased the attention of the remaining participants, or both simultaneously. This uncertainty is especially important given the sample we utilized in this study. Experienced crowdworkers will have developed their own mental models in how to avoid carelessness detection as well as which studies to avoid entirely due to the potential lack of compensation should they not fulfill the researcher’s requirements for answers. As such, comparative research in alternative populations, for example a student population, is still necessary to understand the precise effects of active warning statements. In addition, future research should investigate this by controlling for active engagement when providing warning statements. Given the relatively small effect of the active warning statement, adding one or multiple required open questions might be equally effective in deterring and detecting careless responses, similar to Brühlmann et al. (2020).

Taken together, our results show that merely presenting a warning statement to participants on MTurk is not an effective carelessness prevention method. Increasing the engagement with the warning statement by requiring participants to copy down the statement into a text area led to several statistically significant improvements in terms of consistency and variability of responses. This implies that the current standard practice of implementing warning statements is ineffective and novel methods to prevent and deter carelessness, such as our active warning condition, are needed.

## Limitations and future directions

The main limitation of this study is that the participants were recruited from MTurk. MTurk is a frequently used service in online research, and several works have studied carelessness with participants from this platform. However, alternative services may provide better quality responses (e.g., Prolific, 2022, see Peer et al., 2022 for a recent comparison).

Further, it is important to note that in line with the power analysis, there is a real possibility that the present study was underpowered for detecting statistically significant differences between the warning and control conditions. The sample size allowed for detecting effect sizes of the magnitude *d* = 0.216, with a power of 1-beta = .80, so any smaller, but still practically important effect may have not reached statistical significance in this study.

In addition, it is important to note that while the calculation of even-odd consistency was calculated for groups of items belonging to the same facet of the IPIP-NEO, items measuring the same facet were unlikely to appear close together in the survey due to the randomization. This randomization procedure might have depressed the odd-even consistency scores. However, in its original version, the IPIP-NEO presents items in random order (Goldberg, 1999).

For studies running on MTurk, it is frequently recommended (e.g., Keith et al., 2017) that the filter mechanisms be used, which we deliberately decided against to observe more careless responding. It may be that the potential positive effect of the passive warning statement was masked by the sheer number of low-quality responses in all three conditions. Thus, there is a potential to replicate these findings regarding the ineffectiveness of passive warning statements and the potential of active warning statements with different quality-control settings on the platform.

Another limitation is the wording of the warning statement, which was identical to the study by Toich et al. (2021). Studies demonstrating a positive effect of a passive warning used different, arguably less strongly worded, statements. Future research should explore variants of warnings; however, if such a severe statement as the one used in this study is ineffective, it seems unlikely that alternative, less stern, warning statements work. However, while the studied warning was appropriate for MTurk (e.g., similar to Agley et al. 2022), it may not be possible to use such a strongly worded warning, neither passively nor actively, on other participant recruiting platforms because of their terms of service. Such a strongly worded warning might lead to adverse effects, as described by Huang and Wang (2021). Thus, there is a research opportunity to identify informational or warning statements that work in various contexts.

There is a potential to explore more sophisticated deterrence methods than warnings or informational statements in online research. Future research could explore different variants of required engagement to proceed with the study. For instance, it would be interesting to explore whether the content of the warning affected attentive responding or whether the mere act of requiring participants to write a text is enough to reduce careless responding. Further research might also explore other presentations of warnings, such as videos and audio messages, or novel deterrence methods which are currently underexplored.

## Conclusion

In conclusion, the present study examined the effectiveness of warning statements in reducing careless responding in a sample recruited on MTurk. Results showed that while passive warning statements had no effect on a range of nine carelessness measures, active warning statements had a small but statistically significant effect on self-reported diligence and improved indices of psychometric consistency, even-odd consistency, and response variability. However, active warning statements did not significantly reduce the number of participants flagged by attention check items or affect page response time. These findings suggest that requiring participants to actively process warning statements may have a small to medium effect on reducing careless responding.

## Data Availability

All material and data used for this study are available on OSF under https://osf.io/d2vp4/.

## Footnotes

1 http://www.unipark.de

## Code availability

The analysis script used for this study is available on OSF under https://osf.io/d2vp4/.
